# The impact of orphan histidine kinases and phosphotransfer proteins on the regulation of clostridial sporulation initiation

**DOI:** 10.1128/mbio.02248-23

**Published:** 2024-03-13

**Authors:** Iman Mehdizadeh Gohari, Adrianne N. Edwards, Shonna M. McBride, Bruce A. McClane

**Affiliations:** 1 Department of Microbiology and Molecular Genetics, University of Pittsburgh School of Medicine, Pittsburgh, Pennsylvania, USA; 2 Department of Microbiology and Immunology, Emory University School of Medicine, Emory Antibiotic Resistance Center, Atlanta, Georgia, USA; Ohio State University, Columbus, Ohio, USA; Texas A&M University, College Station, Texas, USA

**Keywords:** *Clostridia*, Spo0A, sporulation, histidine kinases, phosphatases

## Abstract

Sporulation is an important feature of the clostridial life cycle, facilitating survival of these bacteria in harsh environments, contributing to disease transmission for pathogenic species, and sharing common early steps that are also involved in regulating industrially important solvent production by some non-pathogenic species. Initial genomics studies suggested that Clostridia lack the classical phosphorelay that phosphorylates Spo0A and initiates sporulation in *Bacillus*, leading to the hypothesis that sporulation in Clostridia universally begins when Spo0A is phosphorylated by orphan histidine kinases (OHKs). However, components of the classical *Bacillus* phosphorelay were recently identified in some Clostridia. Similar *Bacillus* phosphorelay components have not yet been found in the pathogenic Clostridia or the solventogenic Clostridia of industrial importance. For some of those Clostridia lacking a classical phosphorelay, the involvement of OHKs in sporulation initiation has received support from genetic studies demonstrating the involvement of several apparent OHKs in their sporulation. In addition, several clostridial OHKs directly phosphorylate Spo0A *in vitro*. Interestingly, there is considerable protein domain diversity among the sporulation-associated OHKs in Clostridia. Further adding to the emergent complexity of sporulation initiation in Clostridia, several candidate OHK phosphotransfer proteins that were OHK candidates were shown to function as phosphatases that reduce sporulation in some Clostridia. The mounting evidence indicates that no single pathway explains sporulation initiation in all Clostridia and supports the need for further study to fully understand the unexpected and biologically fascinating mechanistic diversity of this important process among these medically and industrially important bacteria.

## INTRODUCTION

The Clostridia, a polyphyletic class of Bacillota (synonym Firmicutes), do not grow under aerobic conditions and most species stain Gram-positive. They have a widespread environmental distribution, including sewage and soil.

The Clostridia are of considerable interest for several reasons. Some Clostridia, such as *Clostridium leptum*, *Clostridium coccoides,* and *Clostridium scindens,* are important members of the normal human intestinal microbiota that promote health (1). By producing potent toxins, other Clostridia such as *Clostridioides difficile, Clostridium perfringens*, *Clostridium botulinum,* and *Clostridium tetani* are important human and animal pathogens. *C. difficile* is classified as an urgent public health threat by the CDC (https://www.cdc.gov/drugresistance/biggest-threats.html#cdiff), causing nearly half a million cases of *C. difficile* infection every year in the United States, resulting in ~15,000 annual deaths (2). In humans and other animals, *C. perfringens* is an important cause of histotoxic infections, including gas gangrene and intestinal infections (3). *C. perfringens* is also responsible for causing nearly 1 million cases of food poisoning annually in the United States, ranking it as the second most common cause of bacterial foodborne disease (4, 5). *C. botulinum* and *C. tetani* cause lethal flaccid or spastic paralysis, respectively, in many animal species, including humans (6). Lastly, several nonpathogenic Clostridia, for example, *Clostridium acetobutylicum* and *Acetivibrio thermocellus,* have biotechnology importance (7, 8) because they produce industrially useful solvents such as acetone, ethanol, and butanol, some of which can be used as biofuels (7–9).

## THE IMPORTANCE OF SPORULATION FOR THE CLOSTRIDIA

A key characteristic of most Clostridia is their ability to form spores. Since these spores are metabolically inert, spore production permits sustained survival of most Clostridia in nutrient-poor environments (10). Upon encountering an environment with improved nutrient availability, these spores can germinate back into vegetative cells (10).

In addition, all clostridial spores possess resistance against factors such as heat, cold, radiation, and chemicals (including disinfectants and food preservatives), which facilitate survival of these bacteria in harsh conditions. By improving survival in the environment, spore resistance similarly facilitates the transmission of Clostridia causing many diseases, including botulism, tetanus, *C. difficile*-associated disease, *C. perfringens*-induced gas gangrene, and *C. perfringens* type F food poisoning (10). Interestingly, the degree of spore resistance properties can sometimes vary among spores produced by different isolates of the same clostridial species. For example, spores made by many *C. perfringens* type F food poisoning strains are dramatically more resistant to heat and other food environment stresses than the spores produced by other *C. perfringens* strains (11), which enhances their survival in improperly cooked foods and thereby should assist foodborne disease transmission. Sporulation is also necessary for the production of *C. perfringens* enterotoxin, which mediates the pathogenesis of food poisoning and nonfoodborne gastrointestinal disease caused by *C. perfringens* type F (12). As will be discussed in more detail later, sporulation and solvent production are often closely associated with the industrially important Clostridia. For example, phosphorylation of the master transcriptional regulator Spo0A is required for both sporulation and solvent production by *C. acetobutylicum* (13).

Clostridial sporulation involves a relatively conserved sequence of gene expression that governs spore development (8, 10). In all endospore-forming species, including Clostridia, the process of sporulation begins with the activation of Spo0A, the master regulator of sporulation (14). However, the genes and mechanisms that control clostridial Spo0A activation and, thereby, the initiation of sporulation are incredibly diverse. This minireview will address recent advances in understanding the initiation of sporulation in different Clostridia, with a focus on the roles of phosphotransfer proteins in this process.

## COMPARISON OF SPORULATION INITIATION BETWEEN CLOSTRIDIA AND BACILLI

As mentioned, all endospore-forming bacteria initiate sporulation through the highly conserved, essential transcriptional regulator, Spo0A (14). Spo0A functions as a response regulator and the DNA-binding domain is activated by phosphorylation at a conserved aspartate residue in the N-terminal receiver domain (15, 16). Upon phosphorylation, SpooA~*P* dimerizes and directly binds to specific promoter regions containing “0A boxes” to regulate sporulation-specific genes, along with additional stationary phase-associated genes (17). The decision to trigger spore formation requires input from multiple factors to coordinate environmental and metabolic cues that are reflected in the Spo0A phosphorylation state (18). *spo0A* mutants of spore-forming bacteria fail to activate sporulation gene programming and, as a result, are asporogenous.

**Fig 1:**
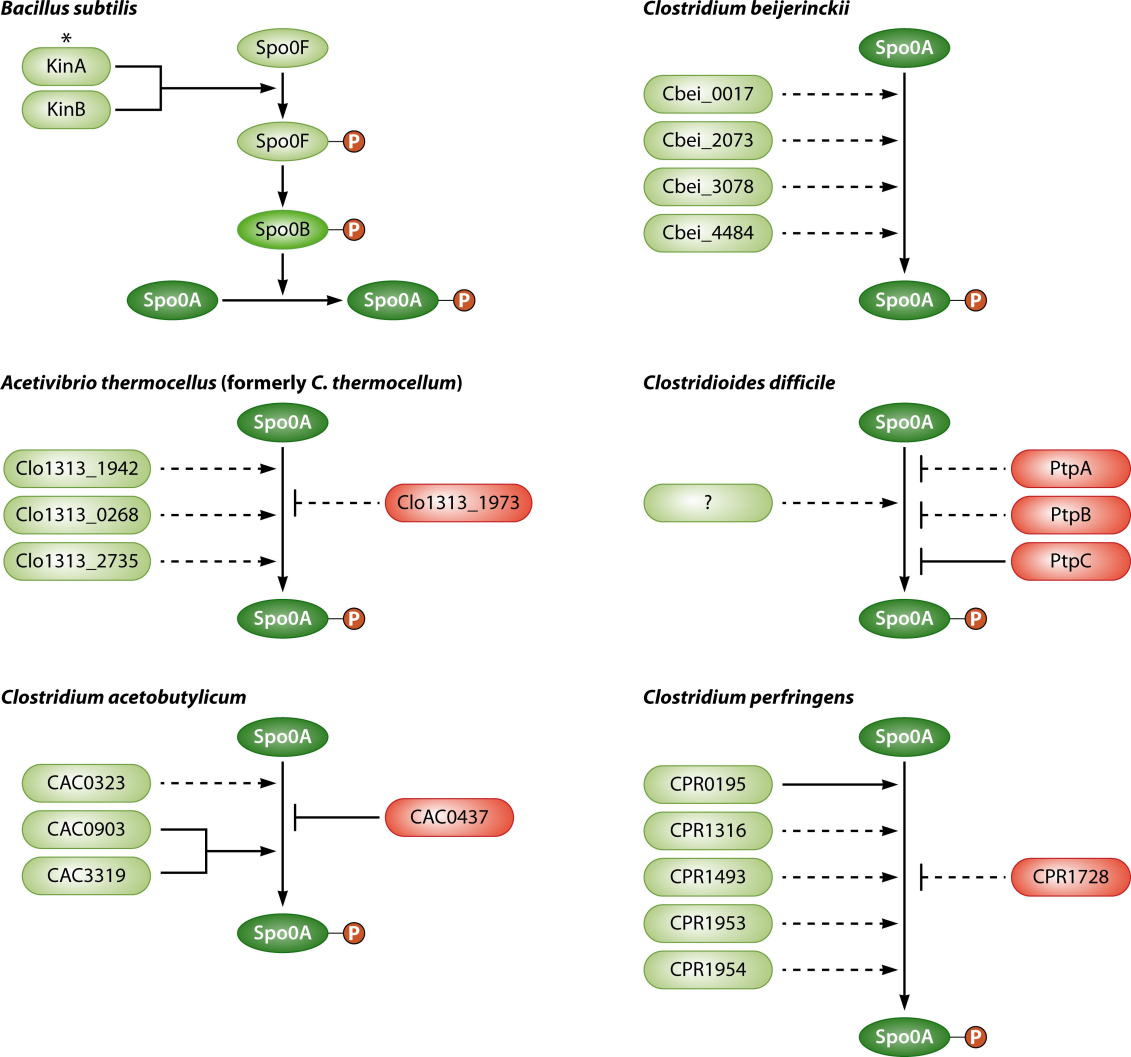
Phosphotransfer proteins affecting the phosphorylation state of the master transcriptional regulator Spo0A in *B. subtilis, A. thermocellus, C. acetobutylicum*, *C. beijerinckii, C. difficile,* and *C. perfringens. B. subtilis* encodes five OHKs, KinA-E, but KinA and/or KinB are the major initiators of sporulation in *B. subtilis* by activating a phosphorelay that controls phosphate flux and leads to Spo0A phosphorylation. *, OHKs KinC-E in *B. subtilis* have minor roles in sporulation under specific genetic conditions and therefore are not shown in this figure. Comprehensive genome sequence analyses initially indicated that Clostridia lack a phosphorelay. More recent studies identified a phosphorelay in some Clostridia (see text), but for the Clostridial species shown in this figure, OHKs (light green) are directly or indirectly implicated in Spo0A phosphorylation. In addition, some OHKs (pink) have been shown to possess dephosphorylation activity and can remove a phosphoryl group from Spo0A-P (see text). Solid lines indicate direct interactions demonstrated *in vitro* while dashed lines represent putative direct interactions that are untested. This figure is updated and modified from reference 8.

Bacilli, including the extensively studied model organism *Bacillus subtilis*, govern Spo0A phosphorylation through an expanded two-component system, known as a phosphorelay (Fig. 1), that controls the flux of phosphate (15). *B. subtilis* encodes five orphan histidine kinases (OHKs), KinA-E, which influence spore formation, along with additional Spo0A-dependent stationary phase processes. The moniker “orphan” refers to histidine kinases encoded by genes not located with genes encoding a cognate response regulator. Upon activation, presumably in response to intracellular and extracellular signals, these histidine kinases autophosphorylate and transfer the phosphoryl group to an intermediate response regulator, Spo0F (19, 20). This phosphoryl group is subsequently relayed to Spo0A through the phosphotransfer protein, Spo0B (15, 21). These consecutive interactions between factors in the phosphorelay are conserved; Spo0F shares significant similarity to a phosphorylatable response regulator receiver domain, and Spo0B is reminiscent of the histidine phosphotransfer domain of histidine kinases (22–24).

Upon starvation, *B. subtilis* initiates sporulation through the histidine kinases KinA and KinB, which are cytosolic and membrane proteins, respectively (19, 25). KinA possesses three PAS (Per-Arnt-Sim) domains, which generally function as molecular sensors. These three KinA PAS domains are important for KinA oligomerization and autophosphorylation (26, 27). Although initially hypothesized to respond to various extracellular and intracellular signals, no known ligand has been identified and verified for these PAS domains. Instead, KinA kinase activity hinges on accumulation to a threshold level within the cell during slower cell growth, often triggered by nutrient deprivation, to contribute to Spo0A phosphorylation (28–30). This is supported by the evidence that KinA activity is regulated by its intracellular concentration (30). Little is known about KinB activation and regulation; however, KinA and KinB contributions to sporulation initiation vary depending upon the growth medium used (31). KinC plays a minor role in sporulation in unique genetic contexts by directly phosphorylating Spo0A (32, 33) but has since been shown to control cannibalism and biofilm formation through Spo0A via the phosphorelay (34, 35), functioning as either a kinase or phosphatase at different growth rates (36). Although an N-terminal PAS domain is necessary for KinC autophosphorylation activity, an activating signal of KinC has yet to be identified (37). KinD is also a bifunctional histidine kinase that promotes biofilm formation (20, 38, 39), similar to KinC. KinD delays sporulation during biofilm formation (38). Interestingly, osmotic pressure from the forming matrix polymer, glycerol, and manganese has been identified as signals to activate KinD activity through a conserved CACHE domain (39–41). Little is known about KinE’s contribution to *B. subtilis* sporulation. Similar to KinC and KinD, KinE appears to play a minor role in sporulation (20). While often generically grouped as the five OHKs required to activate sporulation in *B. subtilis*, KinA-E functions are not redundant, and they integrate diverse growth and environmental signals to influence Spo0A phosphorylation. This complex regulatory pathway calibrates Spo0A activity to control multiple physiological processes. This level of signal input diversity likely exists in other spore-forming bacteria as well.

Although the *B. subtilis* kinases directly interact with Spo0F, which passes the phosphoryl group to Spo0A via Spo0B, orthologs to Spo0F and Spo0B are notably missing in many Clostridial genomes (23, 42, 43). Historically, none of the class Clostridia species were thought to possess a phosphorelay architecture. However, a recent genome neighborhood conservation analysis discovered that many Clostridia encode predicted Spo0F and Spo0B proteins (44). A functional phosphorelay from a class Clostridia member, *Desulfotomaculum acetoxidans*, was experimentally verified, indicating that some Clostridia initiate sporulation through a four-protein phosphorelay similar to Bacilli (44). Yet, the absence of the phosphorelay in many other Clostridia, including the Families Clostridiaceae, Peptostreptococcaceae, and Ruminococcaceae, which contain many pathogenic or non-pathogenic solventogenic species of industrial importance, suggests that Clostridia employ different signaling pathways to initiate sporulation. Several mechanisms for Spo0A activation in the absence of the phosphorelay have been proposed; however, the most likely mechanism is that sporulation-associated sensor OHKs directly phosphorylate Spo0A without intermediate phosphotransfer proteins. Indeed, as detailed below, direct phosphorylation of Spo0A by OHKs has been demonstrated in several Clostridial species (45–48). Still, the possibility remains that a novel phosphorelay, perhaps between multiple histidine kinases, exists to control Spo0A activation in these species.

Finally, the additional early sporulation factors that influence sporulation initiation vary significantly between Bacilli and Clostridia as well (10, 42, 49, 50). In Bacilli, the flux of phosphate to Spo0A is further regulated by two classes of phosphatases that target either Spo0F or Spo0A and anti-kinases. Orthologs to many of these early sporulation factors are encoded in Clostridial species, and unsurprisingly, often exhibit different regulatory functions and mechanisms, likely adapting to the absence of a phosphorelay. While not the focus of this review, it is important to note the significant differences in the ecological niches between and within these two classes. The divergent functions of the early sporulation regulators likely reflect the diversity of environmental cues that trigger sporulation in different species. Supporting this notion, there is a poultry gut-adapted *B. subtilis* strain that does not encode two early sporulation factors, resulting in earlier and higher rates of sporulation (51).

## OVERVIEW OF CLOSTRIDIAL PHOSPHOTRANSFER PROTEINS INVOLVED IN SPORULATION INITIATION

Clostridial phosphotransfer proteins (Table 1) have an assortment of structural architectures that perform a variety of functions in the Spo0A activation pathways of different species. Unfortunately, there are no apparent features, such as the presence of membrane-spanning segments or PAS domains, that allude to the roles of the individual Clostridial phosphotransfer proteins in Spo0A regulation. However, advances in the prediction of specificity residues for histidine kinase-response regulator interactions and function can provide clues to the operation of these proteins as phosphatases or kinases of Spo0A. The clostridial Spo0A proteins share 57%–76% amino acid identity, significantly more so with each other than they do with *B. subtilis* (52). However, analysis of these sporulation phosphotransfer proteins among different Clostridia reveals little structural or sequence similarity for those suspected of directly interacting with Spo0A to regulate sporulation.

**TABLE 1:** Comparison of confirmed and potential sporulation phosphotransfer proteins

Protein	Mutant sporulation^*a*^	Size	Protein structural domains^*b*^^,*c*^	Reference(s) for mutant phenotypes
*B. subtilis*				
KinA	↓	606 aa	3 PAS – HisKA – HATPase	(19, 31)
KinB	↓	428 aa	5 TM – HisKA – HATPase	(25, 31)
KinC	No change	428 aa	2 TM – PAS – HisKA – HATPase	(32, 33)
KinD	↑	506 aa	TM – dCache – HisKA – HATPase	(38, 53)
KinE	ND	738 aa	4 PAS – HisKA – HATPase	
*A. thermocellus*				
Clo1313_0268^*d*^	Spo^-^	239 aa	HisKA – HATPase	(47)
Clo1313_0495	ND	604 aa	dCache – TM – HisKA – HATPase	
Clo1313_0496	ND	616 aa	TM – dCache – HisKA – HATPase	
Clo1313_1711	ND	501 aa	HisKA – HATPase	
Clo1313_1942	Spo^-^	424 aa	REC – HisKA – HATPase	(47)
Clo1313_1973	↑	712 aa	PPBP – HisKA – HATPase	(47)
Clo1313_2735	Spo^-^	387 aa	2 TM – HisKA – HATPase	(47)
*C. acetobutylicum*				
CAC0323	↓	654 aa	7 TM – PAS – HisKA – HATPase	(46, 54)
CAC0437	↑	637 aa	2 PAS – HisKA – HATPase	(46, 55)
CAC0903	↓	683 aa	7 TM – PAS – HisKA – HATPase	(46, 54)
CAC3319	Spo^-^	445 aa	HisKA – HATPase	(46, 54)
*C. beijerinckii*				
Cbei_0017	↓	301 aa	HisKA – HATPase	(56)
Cbei_0807	ND	636 aa	7 TM – PAS – HisKA – HATPase	
Cbei_0808	ND	671 aa	7 TM – PAS – HisKA – HATPase	
Cbei_2073	↓	444 aa	HisKA – HATPase	(57)
Cbei_2504	ND	480 aa	HisKA – HATPase	
Cbei_2732	ND	479 aa	HisKA – HATPase	
Cbei_3078	↓	754 aa	2 PAS – HiskA – HATPase – REC	(56)
Cbei_3079	ND	1024 aa	3 PAS – HisKA – HATPase – REC	
Cbei_4484	↓	568 aa	REC – 2 PAS – HisKA – HATPase	(57)
*C. botulinum*				
CBO0336	ND	615 aa	5 TM – PAS – HisKA – HATPase	
CBO0340	ND	617 aa	5 TM – PAS – HisKA – HATPase	
CBO0780	ND	301 aa	TM – HisKA – HATPase	
CBO1120	ND	477 aa	TM – HisKA – HATPase	
CBO2762	ND	702 aa	2 PAS – HisKA – HATPase	
*C. difficile*				
PtpA	↑	915 aa	8 TM – 3 PAS – HisKA – HATPase	(58)
PtpB	↑	912 aa	8 TM – 2 PAS – HisKA – HATPase	(59)
PtpC	↑	618 aa	PAS – HisKA – HATPase	(59)
*C. perfringens^e^*				
CPR0195	↓	791 aa	8 TM – PAS – HisKA – HATPase	(48, 60)
CPR1055	No change	558 aa	PAS – HisKA – HATPase	(48, 60)
CPR1316	↓	787 aa	7 TM – PAS – HisKA – HATPase	(60)
CPR1493	↓	1086 aa	9 BP/Y-Y-Y – TM – HisKA – HATPase	(60)
CPR1728	↑	571 aa	TM – sCache – HAMP – PAS – HisKA – HATPase	(60)
CPR1953	↓	678 aa	8 TM – HisKA – HATPase	(60)
CPR1954	↓	624 aa	7 TM – PAS – HisKA – HATPase	(60)

^*a*^ ND: not determined.

^*b*^ BP/Y-Y-Y: β-propeller-associated domains; Cache: small molecule recognition; HAMP: histidine kinase, adenyl cyclase, methyl-binding, phosphatase domain; HATPase: histidine kinase-like ATPase; HisKA: His kinase A; PAS: Per-Arnt-Sim sensor; REC: receiver domain; TM: transmembrane domain.

^*c*^ Domains identified during preparation of this review using GenBank sequences of each protein and SMART (Simple Modular Architecture Research Tool) (https://smart.embl.de/).

^*d*^ Truncated product.

^*e*^ Sporulation phenotype of mutants in MDS.

The majority of experimental information on phosphotransfer protein contributions to the initiation of sporulation has been obtained from the Clostridiaceae family, which includes the species *tetani, acetobutylicum, perfringens, beijerinckii*, and *botulinum*. This review will now discuss recent progress in understanding the contributions of phosphotransfer proteins to sporulation initiation for different Clostridia. However, it is worthwhile emphasizing that there are dozens of Families within the Clostridia class for which sporulation, much less the pathways that regulate Spo0A activity, remains completely uncharacterized.

## CONTRIBUTIONS OF OHKs TO SPORULATION INITIATION BY *C. PERFRINGENS*

Since *C. perfringens* lacks an identifiable phosphorelay (48, 61), bioinformatic analyses of the *C. perfringens* type F strain SM101 genome (62) were performed (48), which revealed that SM101 carries seven chromosomal genes encoding putative OHKs. Those putative OHK genes are designated as *cpr0195*, *cpr1055*, *cpr1316*, *cpr1493*, *cpr1728*, *cpr1953*, and *cpr1954*. Automated computational prediction using the PSORTb program suggested that the *cpr0195*, *cpr1316*, *cpr1493*, *cpr1728*, *cpr1953*, and *cpr1954* genes encode putative OHKs with a membrane localization, while CPR1055 is predicted to be cytoplasmic (48, 60). Bioinformatic analyses using the SMART and Interpro programs indicate that all seven of these OHKs possess a histidine kinase-like ATPase (HATPase) domain and a histidine kinase A (HisKA) phosphoacceptor domain, with all but CPR1493 and CPR1953 also possessing a recognizable PAS domain.

Genetic analyses determined that *cpr1953* and *cpr1954* are overlapping genes, sharing 20 nucleotides in common, with the same orientation (60). While these two genes can be co-transcribed as an operon (60), the *cpr1953* null mutant still expresses *cpr1954*, and the *cpr1954* null mutant still expresses *cpr1953* (60). Those observations suggest that *cpr1953* and *cpr1954* can also be expressed from independent promoters.

BLAST analysis (48, 60) indicated that the genes encoding these seven putative OHKs are present in nearly all other genome-sequenced *C. perfringens* strains, except type C strain JGS1495, which apparently lacks the genes encoding CPR1055 and CPR1316. Furthermore, those BLAST analyses suggested these *C. perfringens* OHKs are not encoded by most other Clostridia, including *C. difficile*. A BLAST search had initially indicated *Clostridium novyi* strain NCTC13108 carries genes encoding proteins with high similarity to CPR0195, CPR1493, CPR1728, and CPR1953 but this strain has now been reclassified as *C. perfringens* (https://www.culturecollections.org.uk/products/bacteria/detail.jsp?refId=NCTC+13108&collection=nctc).

Considerable progress was recently achieved in understanding the contributions of these putative OHKs to regulating sporulation and enterotoxin (CPE) production, which is sporulation dependent, by *C. perfringens* type F strain SM101. In 2019, Freedman et al. (48) showed that, in a modified Duncan-Strong sporulation medium (MDS), a *cpr0195* null mutant of SM101 exhibited a ~1,000-fold reduction in sporulation, along with significantly reduced CPE production. In contrast, a *cpr1055* null mutant of SM101 still showed wild-type sporulation and CPE production levels when cultured in MDS. These results indicated that some, but not all, putative OHKs are important for sporulation and CPE production by SM101 under this culture condition. It was also shown that, *in vitro*, the predicted kinase domain of CPR0195 can phosphorylate purified Spo0A. This *in vitro* evidence not only confirms that CPR0195 is a kinase but also supports the hypothesis that some *C. perfringens* OHKs can directly phosphorylate Spo0A, which is the critical first step in initiating sporulation.

A follow-up study (60) then evaluated the contributions of CPR0195 and CPR1055 for regulating sporulation and CPE production by SM101 in a more pathophysiologically relevant incubation condition than MDS. For this purpose, an *ex vivo* model using diluted mouse small intestinal contents (MIC) was developed and shown to support sporulation and CPE production by SM101 (60). Similar to the MDS results, no differences in the levels of sporulation or CPE production were detected between wild-type SM101 and the *cpr1055* null mutant when cultured in MIC. Surprisingly, the *cpr0195* null mutant, which exhibits reduced sporulation and CPE production in MDS, still sporulated and produced CPE at the same levels as wild-type SM101 when cultured in this new MIC model. This finding revealed that environmental conditions profoundly impact the importance of individual *C. perfringens* OHKs for sporulation and CPE production.

Therefore, seven SM101 mutants, each unable to produce a different putative OHK, were compared for their ability to sporulate and produce CPE in MIC vs MDS (60). The results revealed three phenotypes. The *cpr1055* and *cpr1728* null mutants still sporulated and produced CPE at approximately the same levels as wild-type SM101 in both MDS and MIC conditions. In contrast, the *cpr0195*, *cpr1316*, and *cpr1493* mutants showed reduced sporulation and CPE production when cultured in MDS medium but were able to sporulate and produce CPE similarly to SM101 when cultured in MIC. Interestingly, the *cpr1953* and *cpr1954* mutants exhibited negligible sporulation and no CPE production in either MDS or MIC.

SM101 produced ~10^7^ spores/mL when cultured in MDS but ~100-fold fewer spores/mL when incubated in MIC (60). While the SM101 mutants unable to produce CPR0195, CPR1316, or CPR1493 made the same number of spores as wild-type SM101 when cultured in MIC, these mutants showed a 10^2^- to 10^4^-fold reduction in sporulation compared to MDS cultures of wild-type SM101. However, the *cpr1953* and *cpr1954* null mutants exhibited a much greater sporulation defect, producing essentially no (i.e., only ~10/mL) spores whether cultured in MDS or MIC. These results indicated that the CPR1953 and CPR1954 OHKs are virtually essential for sporulation when SM101 is cultured in either MIC or MDS but CPR0195, CPR1316, and CPR1493 OHKs boost sporulation above those MIC sporulation levels when SM101 is cultured in MDS (Fig. 1). The intricate details of these OHK contributions in different incubation conditions require further study.

Bioinformatic analyses (60) detected the presence of a classical DHp histidine phosphotransfer motif in the translated open reading frame sequences encoding all seven putative OHKs. Therefore, an alanine was substituted for the key histidine residue in this phosphotransfer motif for CPR1316, CPR1493, CPR1953, or CPR1954. When plasmids encoding these alanine-substituted OHKs were transformed into their corresponding mutant, there was no increase in sporulation or CPE production, supporting these proteins as histidine kinases.

Using a *spoIIA* operon promoter-driven reporter plasmid, CPR0195, CPR1316, CPR1493, CPR1953, and CPR1954 were shown to function early in sporulation, that is, prior to the production of sporulation-associated sigma factors (60). This result is consistent with the involvement of these four OHKs in Spo0A production and Spo0A phosphorylation. Supporting this contention, Spo0A western blot analyses demonstrated that the *cpr0195*, *cpr131*6, and *cpr1493* null mutants produced less Spo0A protein compared to wild-type SM101 when cultured for 3 h in MDS (60). However, under that same incubation condition, the *cpr1953* and *cpr1954* null mutants, which are almost completely unable to sporulate, made even less Spo0A than the *cpr0195*, *cpr1316* or *cpr1493* OHK mutants. If the incubation period was extended to 5 h in MDS, all mutants produced wild-type levels of Spo0A, except the *cpr1953* null mutant, which still made reduced amounts of Spo0A.

As already mentioned, it was shown (48) that the predicted kinase domain of CPR0195 can directly phosphorylate Spo0A. Similar studies have not yet been performed with the CPR1316, CPR1493, CPR1953, or CPR1954 OHKs. However, studies (60) using Phos-Tag gels indicated that the *cpr1954* kinase mutant has no detectable phosphorylation of Spo0A. Whether CPR1954 directly phosphorylates Spo0A or affects Spo0A phosphorylation through an intermediate remains to be determined, as does the ability of CPR1953 to phosphorylate Spo0A. Collectively, the reduced Spo0A production by the *cpr1953* and *cpr1954* mutants, and the lack of Spo0A phosphorylation for the *cp*r*1954* mutant, can explain the profound defects in sporulation and CPE production by these mutants. The reduction in Spo0A production and phosphorylation for the *cpr1954* mutant may be linked since Spo0A phosphorylation in *Bacillus* spp. increases Spo0A production (63).

Conceivably, CPR1055 or CPR1728 could be phosphatases that, under certain environmental conditions, affect Spo0A phosphorylation levels and thereby modulate (inhibit) sporulation, rather than acting as OHKs to promote sporulation (Fig. 1). Offering limited support for that possibility, the *cpr1728* null mutant sporulated slightly better than SM101 in MDS, although that effect did not reach statistical significance. This mutant also produced slightly more CPE in MDS as assessed by western blotting, but that effect was not quantified.

## CONTRIBUTION OF OHKs TO SPORULATION INITIATION BY *C. DIFFICILE*

*C. difficile* is a gastrointestinal pathogen and is the primary catalyst of antibiotic-associated diarrhea. The symptoms of *C. difficile* infection (CDI) are mediated by two large exotoxins, TcdA and TcdB (64), and range from mild diarrhea and abdominal pain to potentially lethal pseudomembranous colitis. Spores are critical to the *C. difficile* life cycle as they are essential for transmission; therefore, the dormant spore is the infectious form of this bacterium (65). Spores are resistant to many disinfectants used in healthcare settings, which provide persistence in the environment and are often impervious to antibiotic treatment, promoting the reoccurrence of CDI (65–67).

The Spo0A transcriptional regulator in *C. difficile* shares significant similarity to the *B. subtilis* Spo0A amino acid sequence and structure (52, 68). As in *B. subtilis*, the conserved aspartate residue in the N-terminal receiver domain is critical for *C. difficile* Spo0A phosphorylation and dimerization (52). Because *C. difficile* does not possess an identified phosphorelay, it is presumed that any activating histidine kinase would directly bind to and phosphorylate Spo0A, whereas sporulation-associated histidine kinases in *B. subtilis* directly interact with Spo0F. Comparative studies between the receiver domains of *C. difficile* Spo0A and *B. subtilis* Spo0A and Spo0F, coupled with extensive site-directed mutagenesis of conserved residues, revealed that *C. difficile* Spo0A utilizes functionally conserved regions to facilitate interactions with both positive and negative regulators (52).

*C. difficile* encodes several OHKs, three of which share significant homology to KinA and KinB of *B. subtilis* (45): PtpA (CD630_14920), PtpB (CD630_24920), and PtpC (CD630_15790), all named as phosphotransfer proteins (Ptp) for their function in sporulation. PtpA and PtpB are large, transmembrane proteins with three and two predicted PAS domains, respectively. These PAS domains are located intracellularly, although their function remains unknown. PtpC is a cytosolic protein containing a degenerate PAS domain. An early study briefly characterized two of these orphan kinases; they found that a *ptpB* mutant exhibited decreased sporulation, although this mutant was never complemented (45). They also demonstrated that PtpC directly transferred a phosphoryl group to Spo0A *in vitro* (45), suggesting that Spo0A phosphorylation and activation are directly controlled by orphan histidine kinases in *C. difficile*. However, the regulatory roles of PtpB and PtpC were not fully elucidated in this study.

A subsequent study revealed that PtpA inhibits *C. difficile* sporulation, as a *ptpA* mutant hypersporulated (58). Furthermore, the conserved histidine residue required for autophosphorylation and phosphoryl group transfer was critical for PtpA function. Interestingly, additional work revealed that a *ptpB* mutant exhibits increased sporulation in several lab conditions (59), similar to a *ptpA* mutant and in contrast to the initial study (45). Contrary to the initial hypotheses, PtpA and PtpB appear to function primarily as phosphatases that inhibit Spo0A activity and subsequently inhibit spore formation and thus are referred to as phosphotransfer proteins rather than sensor kinases.

PtpA and PtpB phenocopy each other and exhibit identical changes in gene expression (59). A *ptpA ptpB* double mutant displays the same hypersporulation phenotype as the single mutants, suggesting that PtpA and PtpB function in the same regulatory pathway to repress spore formation (59). Neither protein can replace the function of the other, indicating that PtpA and PtpB are nonredundant (59). Surprisingly, unlike PtpA, the conserved histidine residue of PtpB is not necessary for its function (59). It appears that PtpA and PtpB function together, not stepwise, to repress sporulation, suggesting that PtpA and PtpB may only be active as phosphatases when paired as hetero-oligomers. No direct evidence has yet demonstrated that PtpA and PtpB directly bind Spo0A; it remains possible that PtpA and PtpB serve as an endpoint in a serial dephosphorylation pathway. An alternative hypothesis is that PtpA and/or PtpB interact with an intermediate factor(s) to facilitate the dephosphorylation of Spo0A.

PtpA and PtpB affect additional virulence-associated physiological processes in *C. difficile*. Mutants of *ptpA* and *ptpB* produce less TcdA toxin, and a *ptpA* mutant exhibits an attenuated virulence phenotype in the hamster model of *C. difficile* infection (58, 59). PtpA and PtpB also promote motility gene expression, and the *ptpA* mutant is less motile than the parent (58, 59). The PtpA and PtpB regulatory pathway is linked with RstA, a multifunctional regulator that indirectly promotes sporulation and directly represses the expression of motility and toxin genes (58, 59, 69, 70). RstA inhibits the function of Spo0E, a small protein that directly binds to Spo0A and prevents its activation (71). PtpA/B and RstA reciprocally regulate sporulation, toxin production, and motility, which suggests that the activities of the proteins converge on a shared regulatory pathway. However, the regulatory relationship between PtpA/PtpB and RstA remains unclear.

The function of PtpC in early sporulation events has been difficult to discern. While PtpC was shown to directly transfer a phosphoryl group to Spo0A *in vitro*, a *ptpC* mutant exhibits variably increased sporulation on sporulation agar (45, 59). The *ptpC* mutant was complemented with *ptpC* alleles containing site-directed mutations in the predicated residues required for kinase and phosphatase activities, indicating PtpC may not be active on sporulation agar (59). Interestingly, overexpression of *ptpC* in the parent strain resulted in increased spore formation, which was dependent on the conserved histidine residue (59), indicating that at higher intracellular concentrations, PtpC functions to promote Spo0A phosphorylation. Altogether, PtpC appears to function as a dual kinase/phosphatase in response to unknown signals. It is possible that the strong phosphatase activity of PtpA and PtpB masks the effects of PtpC and/or that PtpC functions differently in the host. Additional studies are needed to better understand the contributions of PtpC to Spo0A activation.

There are additional OHKs encoded in *C. difficile*; however, not all of these have roles in regulating early sporulation events. The OHK CD630_13490 (CprK) was found to function as the sensor kinase for the cationic antimicrobial peptide-responsive CprABC system (72). The function of another OHK, CD630_19490, is unknown but does not impact spore formation (59). Another OHK, CD630_05760, now known as RgaS, has recently been identified as the cognate sensor kinase to the orphan response regulator, RgaR (73). RgaR directly activates the transcription of several operons, including *agrB1D1*, which encodes the gene products necessary to produce the AgrD1 quorum-sensing peptide, and *spoZ*, encoding a regulatory small RNA (73, 74). The AgrD1 quorum-sensing peptide accumulates extracellularly and promotes early-stage sporulation through an unknown regulatory pathway (75). SpoZ promotes later-stage sporulation through inhibiting the accumulation of a small protein (73). While RgaS does not directly influence Spo0A activation, the RgaSR two-component system functions at multiple points within the sporulation pathway, including during early sporulation events, to trigger *C. difficile* spore formation.

The presence of multiple, identified Spo0A inactivating factors suggests that mechanisms for deliberate Spo0A phosphorylation occur in *C. difficile* (Fig. 1). However, the mystery of which factor(s) in *C. difficile* are primarily responsible for Spo0A phosphorylation remains unsolved. It seems likely that Spo0A phosphorylation is directly mediated by still unidentified kinases. These potential kinases are traditionally difficult to predict beyond the status of an OHK. Further unraveling how the quorum-sensing peptide, AgrD1, promotes early sporulation may lead to the identification of an activating Spo0A factor. Further delineating the complex genetic pathways and molecular mechanisms by which Spo0A activity is controlled will provide greater insight into the environmental signals that trigger spore formation within the host.

## CONTRIBUTIONS OF OHKs TO SPORULATION INITIATION BY *CLOSTRIDIUM BOTULINUM* AND RELATED SPECIES

*C. botulinum* is a diverse collection of species that has been historically clustered into four genetically distinct groups based on physiological traits (76). All *C. botulinum* produce the characteristic botulinum neurotoxins; however, each of the four groups has a phylogenetically related partner species that is non-toxigenic (77–79). The neurotoxigenic isolates are referred to as *C. botulinum* (Groups I-II) and *C. argentinense* (Group IV). The non-neurotoxigenic counterparts include *C. sporogenes* (Group I), *C. taeniosporum* (Group II), *C. novyi* (Group III), and *C. argentinense, C. subterminale,* or *C. hastiforme* (Group IV). In addition, some *C. baratii* and *C. butyricum* isolates can also make botulinum toxin but are not denoted as *C. botulinum* (80). Of all of these, the only information published on Spo0A post-transcriptional regulation is from the Group I *C. botulinum*, strain ATCC 3502.

In 2006, Wörner et al. scanned the genome in search of OHKs that could serve as *C. botulinum* Spo0A activators (80). This analysis led to the identification of five OHKs: CBO0336, CBO0340, CBO0780, CBO1120, and CBO2762. The investigators cloned and expressed *CBO1120* and *spo0A* from *C. botulinum* in *Bacillus subtilis,* and assessed sporulation outcomes. Heterologous expression of *spo0A_C.b._* alone could not complement the sporulation of a *B. subtilis spo0A* mutant but Spo0A_C.b._ was able to repress expression of the *B. subtilis* Spo0A-regulated gene, *abrB*. Furthermore, they observed that heterologous co-expression of *CBO1120* and *spo0A* was lethal to *B. subtilis*. However, expression of *CBO1120* alone, or a combination of *CBO1120* and an inactive *spo0A* variant, had no effect. From this, they concluded that CBO1120 is likely a direct activator of Spo0A. However, no further studies have been performed to verify the interactions or functions of CBO1120 or the other OHKs. The limited information available on the factors and pathways that regulate Spo0A in the *C. botulinum* groups is likely due to the restrictions on experimentation with neurotoxin producers; however, exploration of these mechanisms in the non-pathogenic relatives or modified *C. botulinum* strains lacking the neurotoxin genes represents an opportunity to advance this field (81).

## CONTRIBUTIONS OF OHKs TO SPORULATION INITIATION BY NON-PATHOGENIC CLOSTRIDIA

There are dozens of families under the order Eubacteriales of class Clostridia, most of which are non-pathogenic species. Compared to the pathogenic Clostridia, there is significantly less known about the post-transcriptional regulation of Spo0A or sporulation in non-pathogenic Clostridia. Only three non-pathogenic species of Clostridia have sporulation kinases or phosphatases that have been characterized genetically or biochemically. These include *Clostridium acetobutylicum*, *Clostridium beijerinckii,* and *Acetivibrio thermocellus* (previously *Clostridium thermocellum*). The studied non-pathogenic Clostridia are important biofuel/solvent generators that were historically employed for the production of the acetone-butanol-ethanol (ABE) solvents. Accordingly, most of what is known about the function of their genes is in the context of their use as industrial producers of compounds. In this section, we describe the current state of research for the three species with experimental evidence for Spo0A regulation: *C. acetobutylicum, C. beijerinckii,* and *A. thermocellus*.

### Clostridium acetobutylicum

*C. acetobutylicum* has been employed for industrial solvent production for over a century. Consequently, there has been significant progress in understanding how *C. acetobutylicum* generates solvents and how to improve the solventogenesis process. It was understood more than 40 years ago that solvent production is closely tied to the activation of sporulation in Clostridial producer species (82–84). Research later verified that Spo0A is an important regulator of both sporulation and solvent production in Clostridia, including *C. acetobutylicum* (85–87). Given the importance of Spo0A in solventogenesis, investigators pursued the identification of factors that directly control Spo0A activity, including phosphotransfer proteins.

The genome of *C. acetobutylicum* (ATCC 824) encodes five OHK/phosphotransfer proteins, four of which affect sporulation (CAC0903, CAC3319, CAC0323, and CAC0437) and one that does not (CAC2730) (46). Of the four influential factors, CAC323, CAC0903, and CAC3319 promote sporulation, as null mutants of these genes demonstrated modest reductions in sporulation (46, 54). However, a *CAC0437* null mutant had increased spore formation, suggesting that it functions as a phosphatase that deactivates Spo0A, rather than as a Spo0A kinase (46, 55). CAC0437, CAC0903, and CAC3319 demonstrated the ability to transfer phosphoryl groups with Spo0A *in vitro*, providing additional support for their roles as direct regulators of Spo0A activity (44, 46). Based on the phenotypes of selected double mutants and the evidence for their direct interaction with Spo0A, Steiner et al. proposed a model for Spo0A regulation that inferred three pathways for Spo0A regulation: (i) CAC0903-CAC3319 phosphorylation of Spo0A, (ii) CAC0323 phosphorylation of Spo0A, and (iii) CAC0437 dephosphorylation of Spo0A (46). However, a subsequent study found that a CAC3319 null mutant was unable to form spores (54), which disrupts the prior model. Investigators were unable to purify active CAC0323, so its ability to directly impact Spo0A phosphorylation remains uncertain. Some of these phosphotransfer proteins may act together in pathways to regulate Spo0A (Fig. 1); however, additional experimentation is required to sort out their specific roles and epistatic hierarchies.

### Clostridium beijerinckii

*C. beijerinckii* is an important solventogenic species of interest as a biofuel producer. Because phenotypic traits were initially used to classify species, many *C. beijerinckii* were historically misclassified as *C. acetobutylicum*, and it was initially thought that regulation of sporulation and solventogenesis were similar between these species. However, genomic comparisons revealed extensive differences in the genomes of these two *Clostridium*, while transcriptional analyses have shown significant differences in their regulation (88–90). The *C. beijerinckii* (strain NCBI 8052) chromosome encodes more than a dozen OHKs, though only a subset was recently examined for a role in sporulation. In 2020, Xin et al*.* evaluated several *C. beijerinckii* OHKs for similarity to the *C. acetobutylicum* Spo0A kinases (57). Based on sequence alignment profiles, they investigated six OHKs: Cbei1553, Cbei2073, Cbei2087, Cbei2435, Cbei4484, and Cbei4925. They reported that null mutants in only two of these genes, *Cbei2073* and *Cbei4484,* formed fewer heat-resistant spores, while deletions in the other four genes had no significant phenotypes. Cbei2073 is noted as having a similar structure and phenotype to the CAC3319 kinase of *C. acetobutylicum* but Cbei4484 is unlike any characterized sporulation phosphotransfer protein. Cbei4484 is predicted to contain the HisKA and HAPTase domains typical of sporulation kinases, but in addition, it includes a receiver domain that is typical of response regulators. The hybrid HK-RR structure of Cbei4484 suggests that this protein may be able to send and receive phosphoryl groups in a complex regulatory arrangement that could include a phosphorelay.

In 2023, Humphreys and colleagues found that sub-culturing mutants for solvent production selected for variants with sporulation defects (56). Through genome sequence analyses, they identified mutagenic “hot spots” in the chromosomes of affected isolates occurring within *spo0A* and the predicted OHKs *Cbei0017* and *Cbei3078*. When assessed for sporulation, the null mutants in both *Cbei0017* and *Cbei3078* demonstrated several log decreases in spores formed, suggesting that they are positive effectors of Spo0A activity. Cbei0017 appears to be a conventional histidine kinase, similar to Cbei2073. But like Cbei4484, Cbei3078 encodes both kinase and receiver domains, suggesting that it may function as a hybrid sensor-receiver, with a more complex signaling role than noted in previously characterized Clostridia. In addition, *C. beijerinckii* encodes at least six other predicted OHKs with similarity to sporulation kinases that have not been examined for sporulation regulatory functions: Cbei0807, Cbei0808, Cbei2160 (hybrid HK-receiver), Cbei2504, Cbei2732, and Cbei3079. Further experimentation, including epistasis analyses and *in vitro* phosphotransfer studies, is necessary to determine the pathways and order of operations for signaling through these factors.

### *Acetivibrio thermocellus* (formerly *Clostridium thermocellum*)

*A. thermocellus* is a soil-dwelling member of the Oscillospiraceae family that is best characterized by its ability to generate bioethanol from cellulose (91). What is known about the kinases or phosphatases that influence *A. thermocellus* sporulation stems from a 2014 study of putative OHKs of strain DSMZ 1313 (47). Using a combination of domain predictions and homology to the *C. difficile* kinase CD2492 (PtpB), the authors identified six predicted sporulation OHKs: Clo1313_268, Clo1313_0495, Clo1313_1711, Clo1313_1942, Clo1313_1973, and Clo1313_2735. They were able to generate null mutations in each of these genes except *Clo1313_0495* and *Clo1313_1711*. Sporulation tests revealed that *Clo1313_268*, *Clo1313_1942*, and *Clos1313_2735* mutants produced no detectable heat-resistant spores, while the Clo1313_1973 mutant generated more spores than the wild-type. Double mutants in *Clo1313_1973* paired with mutants for each of the other genes resulted in wild-type sporulation, suggesting that the function of Clo1313_1973 is dominant to the other factors. Also, the over-expression of *Clo1313_0268, Clo1313_1942,* or *Clo1313_2735* could complement sporulation in any of these mutants, indicating some redundancy in their functions.

Like *C. beijerinckii,* the sporulation phosphotransfer proteins of *A. thermocellus* have diverse structural domains including a hybrid HK-receiver (Clo1313_1942), a predicted periplasmic binding domain (Clo1313_1973), and conventional histidine kinases (Clo1313_0268 and Clo1313_2735); however, none of the examined factors contain defined PAS domains, which are often found in sporulation-associated histidine kinases/phosphotransfer proteins. In addition to the unexplored predicted kinases *Clo1313_0495* and *Clo1313_1711,* the kinase *Clo1313_0496* is predicted to be in an operon with *Clo1313_0495* and may also contribute to sporulation. Given the number of uncharacterized putative sporulation kinases in *A. thermocellus*, it is possible that multiple sporulation initiation pathways or complex regulatory circuits remain to be discovered in this organism.

## SUMMARY

Recent research has revealed great diversity in Spo0A activation pathways used by different Clostridia. Contrary to previous dogma, it is now apparent that some Clostridia encode functional components of the *Bacillus* phosphorelay, but components of that classical phosphorelay have not yet been identified in the pathogenic Clostridia or the nonpathogenic, industrially important solventogenic species. The identification of kinases with receiver domains in some Clostridia opens the possibility that those bacteria may possess a novel phosphorelay for Spo0A phosphorylation. However, the previous hypothesis that Spo0A is directly phosphorylated by OHKs remains the most viable explanation for most of the pathogenic Clostridia and at least some Clostridia with biotechnology importance.

Considering these points, the diversity in Spo0A activation pathways, the variability in kinase/phosphatase use, the structural dissimilarities of the phosphotransfer proteins, and the diversity of the ecological niches inhabited by members of the Clostridia, it is apparent that incredible variability in Spo0A phosphoregulation exists among this class of bacteria. Consequently, no one model can explain Clostridial sporulation initiation; the remarkable diversity in factors that regulate Spo0A among different Clostridia make it difficult to even predict the proteins that directly interact with Spo0A or integrate signals to regulate the onset of sporulation. Therefore, despite much recent progress, further research is needed to continue addressing mechanisms of Clostridial Spo0A phosphoregulation that initiates sporulation, a topic that retains significant relevance for Clostridial pathogenesis and exploitation of Clostridia for industrial purposes.
